# Childhood asthma, inhaled corticosteroid exposure, and risk of cataract in adulthood: a register-based study

**DOI:** 10.1007/s10792-025-03586-3

**Published:** 2025-05-27

**Authors:** Osman Savran, Daniella Bach-Holm, Jens Christian Nørregaard, Line Kessel, Charlotte Suppli Ulrik

**Affiliations:** 1 Department of Ophthalmology, Copenhagen University Hospital Rigshospitalet, Glostrup, Denmark; 2 Department of Respiratory Medicine, Copenhagen University Hospital-Hvidovre, Hvidovre, Denmark; 3 Department of Clinical Medicine, University of Copenhagen, København, Denmark; 4 Øjenklinikken Diakonissestiftelsen, Frederiksberg, Denmark

**Keywords:** Childhood asthma, Cataract risk, Inhaled corticosteroids, Registry-based study, Long-term effects, Dose–response

## Abstract

**Background:**

Cataract is the leading cause of blindness worldwide, with corticosteroid treatment being a known risk factor. The long-term impact of childhood asthma and, particularly, inhaled corticosteroid (ICS) use in adulthood on cataract development remains unclear.

**Methods:**

This register-based study investigated the prevalence and risk of cataract in Danish adults diagnosed with childhood asthma who, between 1950 and 1979, spent four months at an asthma care facility in Kongsberg, Norway. Follow-up was conducted in 2021 using Danish national health registries (2006–2018). These individuals were compared to an age- and sex-matched control group with no history of obstructive airway disease. Participants were stratified by ICS treatment duration and daily dose. Conditional logistic regression was used to assess associations.

**Results:**

The study included 1394 adults with childhood asthma and 1394 controls (mean age 63 years; 43% female). Cataract prevalence was 6.1% in the childhood asthma cohort versus 4.3% in controls (*p* = 0.03). Compared to controls, individuals with childhood asthma had increased odds of cataract (OR 1.47, 95% CI 1.04–2.08, *p* = 0.03). Among those treated with ICS, the odds were higher (OR 1.75, 95% CI 1.19–2.57, *p* < 0.01), with the risk increasing in proportion to ICS dose and treatment duration. No significant difference in cataract risk was found between individuals with childhood asthma who did not receive ICS and controls (OR 1.12, 95% CI 0.69–1.79, *p* = 0.65).

**Conclusions:**

Childhood asthma diagnosis alone was not associated with increased cataract risk. However, among those treated with ICS in adulthood, there was a significantly elevated risk, which increased with higher doses and longer treatment durations.

## Introduction

Cataract is the primary cause of blindness worldwide, affecting millions. It is primarily addressed through surgical intervention [1]. Approximately 80 million people over 50 years of age suffer from visual impairment due to cataract [2]. Asthma and the use of inhaled corticosteroids (ICS) have been associated with an increased risk of cataract development, although the association is less well established than for oral corticosteroids [3–5].

Asthma is a chronic respiratory disease characterized by persistent airway inflammation, variable airflow obstruction, and symptoms, such as wheezing, shortness of breath, cough, and chest tightness [6]. It affects nearly 250 million people globally [7]. ICS are the cornerstone of asthma treatment because they effectively reduce inflammation in the airways [6].

A few studies have demonstrated an association between increased risk of cataract development and other types of corticosteroids. The study by Thorne et al., published in the last decade, found that increasing doses of eye-drops containing corticosteroids were associated with increasingly higher risk of cataract development [8]. A cross-sectional study by Delcourt et al. found increasingly higher risk of cataract development with increasing systemic corticosteroid exposure in patients with asthma [9]. Several studies have assessed the association between cataract development and ICS exposure in patients [10–13]. The study by Cumming et al. found that increasing ICS doses were linked to cataract development, with a nearly threefold risk with at least five years of ICS dosage [10]. However, the relatively small number of asthma patients (*n* = 122) raised doubts about the validity of these findings regarding ICS treatment duration and cataract development. The retrospective cohort study by Jick et al. indicated an increased risk of cataract in patients receiving at least 40 ICS prescriptions [11]. However, this study did not include the risk associated with ICS duration. Additionally, the study by Garbe et al. found that cataract extraction risk was linked to ICS exposure in a three year period, but lacked information on drug use before the age of 65 [12]. None of these studies have investigated cataract risk in adults with a history of childhood asthma.

Our study, therefore, fills an important gap in knowledge by examining cataract risk in adults with a history of childhood asthma. We hypothesized that individuals with a history of childhood asthma, especially those treated with ICS, would have a higher risk of cataract in adulthood compared to individuals without a diagnosis or treatment for obstructive airway disease in adulthood. By comparing adults with a history of childhood asthma to those without asthma, we aimed to elucidate the long-term impact of childhood asthma and, especially, its treatment with ICS on cataract development.

## Material and methods

### Childhood asthma cohort

Between 1950 and 1979, approximately 5000 Danish children with asthma aged 3–13 years, referred to as the Kongsberg cohort, were referred by physicians at the former Queen Louise’s Children’s Hospital in Copenhagen to an asthma care facility in Kongsberg, Norway [14]. Clean and dry Norwegian mountain air was believed to improve their asthma symptoms. All children were diagnosed with asthma by physicians before their stay in the asthma care facility.

**Fig. 1:**
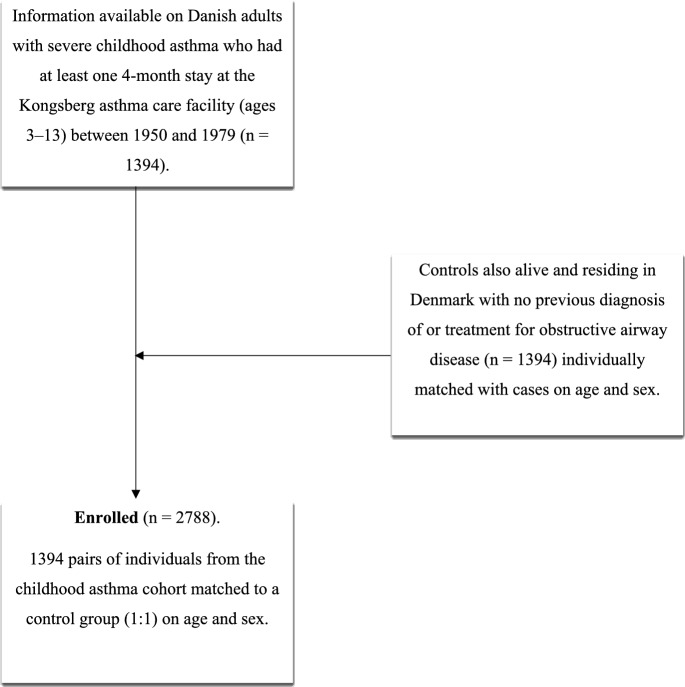
Flowchart of the selection process in the study

Index cards and registration lists were available for all groups of children who had stayed in Kongsberg. These data were linked to each individual’s identification number in three steps: (1) digitization of data by entering individuals’ names and dates of birth from the index cards and registration lists; (2) extraction of personal identification numbers from the Civil Registration System for individuals with matching names and dates of birth; and (3) matching of personal identification numbers with the names and birth dates in individuals diagnosed with childhood asthma using data from the Danish Health Data Authority. Data was collected in 2021 for individuals diagnosed with childhood asthma and a control group. The selection process for the participants in this study is illustrated in Fig. 1.

### Control group

The characteristics of the individuals diagnosed with childhood asthma were compared to a control group matched for age (year of birth) and sex (1:1). Individuals were eligible for the control group provided they had no previous record of hospital contact with a diagnosis of obstructive airway disease, such as asthma or Chronic Obstructive Pulmonary Disease (COPD), and no previous prescriptions for medication for obstructive airway disease. Statistics Denmark conducted searches in nationwide health registers, specifically the National Patient Registry, for ICD-10 codes J44, J45, and J46, to exclude individuals with a registered diagnosis of COPD and/or asthma in secondary care. Additionally, individuals with no current or previous prescriptions were identified through the ATC code R03 (drugs for obstructive airway disease) in the Danish National Prescription Registry and excluded until the index date of June 2022. Search methodology for data retrieval in Danish health registers for the control group was carried out as for the individuals diagnosed with childhood asthma.

### Study design and data sources

This register-based study on individuals diagnosed with childhood asthma included data from several comprehensive Danish national health registries from Statistics Denmark: the National Patient Registry (covering all inpatient hospital contacts, including every diagnosis since 1977 and all outpatient visits since 1995, with mandatory registration by the treating health care professional), the National Prescription Registry (including all prescriptions redeemed at pharmacies since 1995 using anatomical therapeutic chemical [ATC] codes), and the Civil Registration System (including demographic information). Data combination was performed by Statistics Denmark using individuals’ personal identification numbers and the data were pseudonymized before analysis.

### Definitions

Cataract development: A cataract diagnosis was defined as a hospital contact with a primary diagnosis of ICD-10 codes H259-H269.

ICS treatment: ICS doses were calculated as average daily ICS dose exposure during the study period based on redeemed prescriptions. ICS doses were reported as equipotency of budesonide as follows: < 200 mcg/day, 200–400 mcg/day, > 400–800 mcg/day, and > 800 mcg/day doses [6, 15]. ICS treatment duration was categorized as redeeming at least one ICS prescription in less than 4 consecutive years, 5–10 consecutive years, or more than 10 consecutive years.

### Confounders

To ensure the validity of the results, we adjusted for diabetes and corticosteroid use in the logistic regression analysis. Diabetes was identified from the National Patient Registry using ICD-10 codes E10–E14, which represent different forms of diabetes mellitus, with the presence of any code indicating a diagnosis of diabetes. Corticosteroid use, a known cataract risk factor, was accounted for using ATC codes: H02AB07/H02AB06 (systemic corticosteroids), D07 (topical), R01AD (nasal), and S01BA/S01BB/S01CA/S01CB (ophthalmic). These codes signified prescriptions for corticosteroids via different routes. Adjusting for the occurrence of these ICD-10 and ATC codes helped control for the confounding effects of these factors in the analysis.

### Statistical analysis

The individuals diagnosed with childhood asthma and control group demographics were assessed using the most recent dataset from 2018, provided by Statistics Denmark. The extracted register data were reported as mean ± standard deviation (SD). Categorical variables are presented as numbers and percentages. Cataract incidence, ICS daily dose, and treatment duration were assessed retrospectively in the years 2006–2018 and presented as absolute and relative frequencies.

Conditional logistic regression was used to assess the risk of cataract development based on daily ICS doses and treatment duration of ICS in individuals diagnosed with childhood asthma. Conditional logistic regression was also used to assess the risk of cataract development in both the childhood asthma cohort and the control group. Results are reported as odds ratios (OR) with 95% confidence intervals (CI). Regression analyses were adjusted for age, sex, diabetes, and use of systemic, topical, nasal, and eye drop containing corticosteroids.

Statistical significance was set at *p* < 0.05. Data were analysed using the R Statistics 3.61 software (R Foundation for Statistical Computing, Vienna, Austria).

## Results

### Comparison of childhood asthma cohort and matched control group

A total of 1394 Danish individuals with a history of childhood asthma and a previous stay at an asthma care facility in Kongsberg, Norway, were compared to a matched control group (mean age 62.7 years, 43% females). During the assessment period (2006–2018), 6.1% of individuals diagnosed with childhood asthma had cataract compared to 4.3% in the control group (*p* = 0.03) (Table 1).

**Table 1:** Characteristics of individuals diagnosed with childhood asthma (*n* = 1394) compared to age and sex matched controls (*n* = 1394) with no previous recorded diagnosis or treatment for obstructive airway disease

Variable	Cases *n* (*n* = 1394)	Controls *n* (*n* = 1394)	*p*-Value
Mean age (years, SD)	62.7 (8.3)	62.7 (8.2)	NA
Sex (no, %)			
Female	603 (43.3)	603 (43.3)	NA
Cataract (no, %)	85 (6.1)	60 (4.3)	0.03

Demographic characteristics were obtained from recent data reports from national health registers. Accumulated cataract prevalence was obtained from national health registers during the assessment period of 2006–2018

Risk of cataract was significantly higher in individuals diagnosed with childhood asthma than in the control group (OR 1.47, 95% CI: 1.04–2.08, *p* = 0.03) (Table 2). Subgroup analysis indicated that individuals diagnosed with childhood asthma receiving ICS treatment (*n* = 841) had a significantly higher OR for cataract (OR 1.75, 95% CI: 1.19–2.57, *p* < 0.01) compared to those in the control group. In contrast, no significant increase in the OR for cataract was observed (OR 1.12, 95% CI: 0.69–1.79, *p* = 0.65) in individuals diagnosed with childhood asthma receiving no ICS treatment (*n* = 553) compared to the control group (Table 2).

**Table 2:** Conditional logistic regression model for the assessment of cataract risk in individuals diagnosed with childhood asthma (*n* = 1394) compared to age and sex matched controls (*n* = 1394) with no previous recorded diagnosis or treatment for obstructive airway disease

	*n*	Crude OR	Adjusted OR	Adjusted *p*-value
Controls	1394	Ref	Ref	
Cases	1394	1.44 (1.03–2.03)	1.47 (1.04–2.08)	0.029
Excluding those not treated with ICS				
Controls	1394	Ref		
Cases	841	1.62 (1.11–2.35)	1.75 (1.19–2.57)	0.005
Excluding those treated with ICS				
Controls	1394	Ref		
Cases	553	1.86 (0.75–1.88)	1.12 (0.69–1.79)	0.645

The analysis was adjusted for age, sex, diabetes, and systemic, topical, nasal, and eye drop containing corticosteroids. Information on cataract prevalence was obtained from national health registers during the assessment period of 2006–2018

### ICS treatment and cataract risk in the childhood asthma cohort

Based on the available information, including the nationwide register of prescription redemptions, 841 individuals (60.3%) in childhood asthma cohort were treated with ICS. Out of the entire childhood asthma cohort (*n* = 1394), 57 individuals (4.1%) who received ICS treatment developed cataract. Of those who developed cataracts during the assessment period, 63.2% (36 out of 57) had been treated with ICS for more than 10 years. In contrast, 51.9% (407 out of 784) of individuals who received ICS but did not develop cataracts had been treated for less than 10 years (see Table 3).

**Table 3:** Inhaled corticosteroid (ICS) treatment and cataract development in individuals diagnosed with childhood asthma (*n* = 1394)

	*n* (%)	Mean	SD
No cataract + no ICS treatment	525 (37.7)		
No cataract + ICS treatment duration (years)	784 (52.2)		
< 4	186 (23.7)		
5–10	221 (28.2)	2.13	1.14
> 10	377 (48.1)	11.26	2.44
No cataract + ICS daily doses (mcg/day)	784 (52.2)		
< 200	624 (79.6)		
200–400	132 (16.8)		
> 400–800	24 (3.1)		
> 800	4 (0.5)		
Cataract + no ICS treatment	28 (2.0)		
Cataract + ICS treatment duration (years)	57 (4.1)		
< 4	4 (7.0)		
5–10	17 (29.8)	2.00	1.41
> 10	36 (63.2)	11.25	2.91
Cataract + ICS daily doses (mcg/day)	57 (4.1)		
< 200	43 (75.4)		
200–400	10 (17.5)		
> 400–800	4 (7.0)		
> 800	0 (0)		

Information on ICS treatment and cataract prevalence was obtained from national health registers during the assessment period of 2006–2018. The ICS daily dose values (e.g., ‘ < 200’, ‘200–400’, ‘ > 400–800’) and ICS treatment durations (e.g., ‘ < 4’, ‘5–10’, ‘ > 10’) correspond to dose and year intervals, respectively

The OR of developing cataract was 2.2 (95% CI: 1.2–4.1, *p* = 0.02) for those with 5–10 years of ICS treatment and 2.5 (95% CI: 1.5–4.1, *p* < 0.01) for those with more than 10 years of ICS treatment compared to those with less than 4 years of ICS treatment. Furthermore, the OR for cataract development was 1.1 (95% CI: 0.6–2.4, *p* = 0.73) for individuals with an average ICS daily dose between 200–400 mcg and 5.5 (95% CI: 1.1–28.9, *p* = 0.03) for those with an average ICS daily dose of more than 400–800 mcg compared to those with an average ICS daily dose of less than 200 mcg (Table 4).

**Table 4:** Conditional logistic regression model for the assessment of cataract risk with the number of prescriptions and treatment duration of inhaled corticosteroids (ICS) in individuals diagnosed with childhood asthma (*n* = 1394)

	Crude OR (95% CI)	Adjusted OR (95% CI)	Adjusted *p*-value
ICS daily doses (mcg/day)			
< 200	Ref	Ref	
200–400	1.09 (0.53–2.22)	1.14 (0.55–2.38)	0.73
> 400–800	2.66 (0.57–12.36)	5.50 (1.12–28.97)	0.03
ICS duration (years)			
< 4	Ref	Ref	
5–10	1.71 (0.93–3.14)	2.17 (1.16–4.05)	0.02
> 10	2.12 (1.29–3.47)	2.47 (1.49–4.11)	< 0.01

The analysis was adjusted for age, sex, diabetes, and systemic, topical, nasal, and eye drop containing corticosteroids. Information on cataract prevalence and ICS therapy was obtained from national health registers during the assessment period of 2006–2018. The ICS daily dose values (e.g., ‘ < 200’, ‘200–400’, ‘ > 400–800’) and ICS treatment durations (e.g., ‘ < 4’, ‘5–10’, ‘ > 10’) correspond to dose and year intervals, respectively

### Sensitivity analysis

#### Adjustment for socioeconomic status

We performed a sensitivity analysis adjusted for socioeconomic status (SES) to account for potential confounding factors related to access to healthcare and overall health management. SES was assessed using educational level (defined as either low (i.e. basic (compulsory school), upper secondary, or vocational) or high educational level (bachelor’s or master’s degree)) from the Civil Personal Registration registry, Statistics Denmark. After adjusting for SES, the association between cataract risk of ICS treatment in individuals diagnosed with childhood asthma remained significant (OR 1.45, 95% CI: 1.05–2.02).

## Discussion

Several studies have confirmed the relationship between corticosteroid treatment and cataract development in patients with asthma. However, this is the first study to examine cataract development in individuals diagnosed with childhood asthma compared to a control group. Over half of the cohort was treated with inhaled corticosteroids (ICS) as adults, and we found a dose–response relationship where higher ICS treatment duration and ICS daily dose were associated with an increased risk of cataract development.

The development of cataract, particularly posterior subcapsular cataract (PSC), in patients treated with ICS varies between children and adults based on available studies. In children, one might assume a higher sensitivity to cataract development because of the developing tissues [16, 17]. However, observational and randomized controlled studies indicate that low to medium doses of ICS do not significantly increase the risk of cataract in children, adolescents, and young adults [18–21]. Multiple non-randomized studies have suggested that long-term ICS therapy in children does not significantly increase the risk of cataract [18–20]. Furthermore, a randomized controlled study that followed children aged 4–6 years who received at least 898 mg cumulative ICS dose found no significant increase in cataract risk by the time these children reached 15–26 years of age [21].

In adults, particularly older adults, there is an increased baseline risk of cataract due to aging [22]. Studies on adults consistently show a strong association between high-dose ICS treatment and the development of cataract, including PSCs [10–12, 23]. However, limitations in study design, such as reliance on self-reported diagnoses or small sample sizes, have raised questions about the robustness of these associations. For instance, one study found a significant cataract risk with high-dose (> 1000 µg) daily ICS [13]; however, it was based on elderly subjects with limited information on drug use and no confirmation of cataract diagnoses through clinical records. Another study reported an increased cataract risk with corticosteroid use, particularly systemic corticosteroids, but with a relatively small sample size [24]. A recent cross-sectional study suggested a relationship between cataract and asthma in an older population, but its reliance on self-reported diagnoses raised doubts about a definitive association [5]. Our results align with those of previous studies by Cumming et al., Garbe et al., and Jick et al., who also found an increased risk of cataract, even with less ICS exposure [10–12]. Cumming et al. found an increased risk of cataract in a cross-sectional study of 3313 individuals identified in the Blue Mountain Eye Study [10]. High doses of ICS throughout life are associated with an increased risk of cataract. However, in contrast to our study, the interpretation of this study was limited by the small number of patients using ICS (*n* = 327), the use of an individual’s recall to assess drug history, and missing information on drug history and potentially confounding variables. In a large case–control study of 25,545 adults aged 70 years and older, Garbe et al. demonstrated that the use of ICS for more than 3 years increased the risk of cataract extraction (OR 3.06, 95% CI 1.53–6.13) [12]. Although drug history was described in detail in this study, the database provided information on only a limited age range of patients older than 65 years as opposed to our study. A recent register-based cohort study from the NORDSTAR collaboration, which includes comprehensive nationwide register data on all asthma patients from four Nordic countries, investigated the relationship between ICS use and corticosteroid related adverse events, including cataract (defined by ICD codes H25, H26, and/or procedure codes CJC00-99, CJD00-99, and CJE00-99) [25]. The study included 529,203 individuals with asthma aged 18 years and older between 2009 and 2019. Researchers assessed current ICS exposure and calculated average daily ICS doses (expressed as budesonide equivalents). Their findings demonstrated that cataract risk increased with higher ICS doses, with the greatest risk observed among patients currently using very high doses (1600 mcg per day).

Overall, despite the theoretical sensitivity in children, evidence shows that those on low to medium doses of ICS do not pose a significantly increased risk of cataract [21]. In contrast, adults, especially those with prolonged exposure to high doses of ICS, demonstrate a marked increase in cataract risk [10–12, 23]. Therefore, while children appear to tolerate the recommended ICS doses without significant cataract risk, adults may need careful dose management and monitoring when undergoing long-term ICS therapy to mitigate the risk of cataract.

Interestingly, individuals diagnosed with childhood asthma who had not received ICS treatment as adults did not have a significantly increased risk of cataract compared to the control group. A likely explanation is that this group represents individuals whose asthma went into remission in adolescence or early adulthood. Previous studies have shown that children diagnosed with asthma without persistent symptoms or allergic sensitization, are more likely to experience remission [26, 27]. This may explain why they did not require ICS in adulthood and why they may not carry the same elevated risk profile for cataract development.

### Strengths and limitations

The present study has several strengths. We conducted a comprehensive analysis by examining cataract risk in individuals with and without ICS treatment within the childhood asthma cohort. This approach allowed us to assess whether ICS treatment was a potential confounder in the association between history of childhood asthma and cataract development in adulthood. Unlike previous research, which often focused on corticosteroid-treated asthma patients with limited exposure periods and insufficient details of drug use or clinical records, our study leveraged extensive national health registries. This provided a thorough description of the childhood asthma cohort and prescription data. Additionally, the inclusion of the childhood asthma cohort in this study offers a unique perspective, enabling us to explore cataract risk in adulthood. Furthermore, sensitivity analyses were performed to ensure the robustness of our findings.

In contrast to previous studies, our study found that individuals who did not receive ICS treatment did not have a significant risk of cataract development compared with the control group. By distinguishing between individuals receiving ICS and those not receiving ICS, we addressed the potential confounding effects of corticosteroid exposure.

Our study has noteworthy limitations. The observational nature of this study introduces inherent limitations, including reliance on historical data and potential biases. Although we identified an association between childhood asthma and cataract development in adulthood, establishing causation requires a different study design. Moreover, the findings may be specific to the Danish population and may not be directly generalizable to other demographic groups. Although we observed individuals up to the age of 63 years, a longer follow-up period might provide insight into cataract risk beyond this age.

The increased cataract risk associated with prolonged ICS use among individuals diagnosed with childhood asthma may be partially attributed to uncontrolled confounding, potentially related to the increased risk of cataracts associated with asthma itself. In addition to corticosteroids, underlying inflammation in asthma has been suggested as a risk factor for cataract development [28]. Although a pathway linking allergic asthma to cataract development has been described, further investigation is needed to explore the potential association between chronic airway inflammation in asthma and cataract risk. Additionally, the inability to adjust for asthma severity due to limitations in available data, such as the lack of information on daily ICS doses, presents a challenge in fully understanding this relationship. Although it was possible to measure and adjust for the effects of some important confounders, no information on other important confounding factors, such as smoking and body mass index, was available. This information was not available on the national health registers.

The method of diagnosing cataract by hospital contact with a primary diagnosis code (ICD-10 codes DH259-DH269) is subject to limitations, particularly the under-reporting of patients who undergo cataract surgery. This issue is compounded by the lack of registration of cataract surgeries by private hospitals and clinics, potentially impacting the accuracy of the data derived from national registries, as reported by Bjerrum et al. [29]. Differences in registration practices between hospital and private settings could lead to disparities in reported prevalence rates, reflecting variations in registration rather than the actual prevalence.

Asthma often coexists with other conditions that may contribute to cataract development, such as allergic conjunctivitis, which is more prevalent in individuals with asthma and can influence cataract formation [30]. Additionally, the inflammatory response associated with asthma and systemic factors like infections and fever, more common in children with asthma, may also play a role [17, 31]. While corticosteroid exposure is a significant factor, it may only be one piece of the puzzle in understanding the relationship between childhood asthma and cataract development.

Furthermore, it is important to consider the diagnostic uncertainty surrounding early-onset asthma. Some of the youngest individuals in our childhood asthma cohort were diagnosed at the age of 3 years. It is possible that transient wheezing disorders or other non-asthmatic respiratory conditions were misclassified as asthma. This diagnostic uncertainty introduces a degree of heterogeneity in the childhood asthma cohort and may dilute the true association between childhood asthma and adult outcomes such as cataract. These considerations emphasize the complexity of interpreting long-term health risks associated with early-life diagnoses based on potentially limited diagnostic criteria.

Our data covers the period from 2006 to 2018. We had no information on the treatment of our childhood asthma cohort when diagnosed with asthma during childhood. Given that some of the individuals diagnosed with childhood asthma were referred to an asthma care facility from the early 1970s until its closure in 1979, it is likely that some individuals might have been treated with ICS for at least a few years coinciding with their stay at the facility. Since most of the individuals diagnosed with childhood asthma currently receive ICS treatment (60.3%), it is reasonable to infer that they have been treated with ICS for many years. Therefore, this cohort may be useful for assessing the association between childhood asthma, ICS treatment, and cataract development in adulthood.

The approximation of 5000 children in the original childhood asthma cohort is based on the referral of groups of approximately 50 children, four times a year, to the Kongsberg asthma care facility from 1950 to 1979. However, it is possible that some children were referred more than once, which could have led to an overestimation of the number of eligible individuals. To obtain information on currently living individuals from the original cohort, names and birth dates were matched to central personal registration numbers through the Danish Health Data Authority. This method could have resulted in the inclusion of individuals who were not previously referred to the Kongsberg asthma care facility but had similar names and birth dates to those on the registration lists. Additionally, some names and birth dates did not match any personal registration numbers, potentially omitting eligible individuals.

A key limitation of our study is the absence of data on smoking status, which is a well-established independent risk factor for cataract [32]. Smoking contributes to oxidative stress and lens damage [33]. Due to the lack of individual-level data on smoking in the Danish national registries, we could not adjust for this variable in our analyses. This residual confounding may have influenced our findings and limits the strength of causal inference regarding ICS use. Future studies should incorporate smoking history and other lifestyle factors to further clarify these relationships.

Finally, since cataracts are primarily managed in the primary care sector, cases diagnosed and managed outside hospital settings may not be effectively captured using our methodology. If cataract diagnosis was listed as a secondary rather than a primary diagnosis code, it might have been missed in our analysis, potentially leading to underestimation of cataract prevalence. Variations in coding practices and healthcare utilization patterns can introduce biases and limitations to the study findings. Therefore, it is essential to acknowledge these potential limitations and to interpret the results with caution.

## Conclusion

This study provides important insights into the relationship between childhood asthma, inhaled corticosteroid (ICS) treatment, and cataract development in adulthood. And there was a clear dose–response relationship, with increased ICS treatment duration and a higher average daily ICS dose in the assessment period being associated with a higher risk of cataract development. Individuals diagnosed with childhood asthma exhibited 1.4 times higher odds of developing cataract than the control group. Notably, individuals diagnosed with childhood asthma who received ICS treatment had a significantly higher risk of cataract development, whereas those who did not receive ICS showed no significant increase in risk compared to the control group. These results highlight the need for careful monitoring of cataract development in patients undergoing ICS treatment and the potential ocular side effects when prescribing ICS. Further research is needed to explore the mechanisms underlying this association.

## Data Availability

No datasets were generated or analysed during the current study.

## Abbreviations

ICS: Inhaled corticosteroids
COPD: Chronic obstructive pulmonary disease
OR: Odds ratio
CI: Confidence interval
PSC: Posterior subcapsular cataract
SD: Standard deviation
SES: Socioeconomic status
ATC: Anatomical therapeutic chemical
ICD: International classification of diseases
mcg: Microgram
